# Does the Adoption of Climate-Smart Agricultural Practices Impact Farmers’ Income? Evidence from Ghana

**DOI:** 10.3390/ijerph19073804

**Published:** 2022-03-23

**Authors:** Wonder Agbenyo, Yuansheng Jiang, Xinxin Jia, Jingyi Wang, Gideon Ntim-Amo, Rahman Dunya, Anthony Siaw, Isaac Asare, Martinson Ankrah Twumasi

**Affiliations:** 1 College of Economics, Sichuan Agricultural University, Chengdu 610000, China; 2020508001@stu.sicau.edu.cn (W.A.); xinxinj@stu.sicau.edu.cn (X.J.); jingyiworkday@126.com (J.W.); siawanthony@stu.sicau.edu.cn (A.S.); twuma2012@stu.sicau.edu.cn (M.A.T.); 2 College of Management, Sichuan Agricultural University, Chengdu 610000, China; ntimamogideon@stu.sicau.edu.cn (G.N.-A.); rahman@stu.sicau.edu.cn (R.D.); asareisaac12@stu.sicau.edu.cn (I.A.)

**Keywords:** climate change, adoption, climate-smart agricultural practices, endogenous switch regression, telasso treatment effect

## Abstract

People’s lives, particularly farmers’, have been affected by extreme weather conditions that have reduced the yield of numerous crops due to climate change. Climate-smart agriculture practices can reduce or eliminate greenhouse gas emissions and have the propensity to increase farm income and productivity. Therefore, the purpose of this study is to ascertain whether CSA practices impact farmers’ income. This study includes all cocoa farmers in the selected districts in the Ashanti Region. The population includes those who live in the six cocoa production villages. The multistage sampling procedure was considered based on the dominants of literature. The study used an endogenous switching regression framework to examine the effects of the adoption of climate-smart agricultural practices (CSAPs) on farmers’ income. While estimating treatment effects, telasso uses lasso techniques to select the appropriate variable sets. The results revealed that gender, farm experience, age, household size, and farm size do not significantly influence the adoption of irrigation and crop insurance. The study revealed a significant positive impact of access to credit on adopting irrigation and crop insurance. The adoption of climate-smart practices has a positive coefficient. This indicates that if all respondents in each region adopts these practices, their income would increase significantly. This study shows that adopting irrigation practices leads to an increase in household income of 8.6% and 11.1%, respectively, for cocoa farmers. Crop insurance has a positive coefficient and is statistically significant on household income, on-farm, and off-farm. This paper shows that climate-smart practices such as crop insurance can positively influence farmers’ income in Ghana. We also conjecture that crop insurance is the most effective and efficient climate-smart practice among the various agricultural practices. The study suggests that access to credit and mass awareness should be compulsory modules coupled with the consistent training of farmers on new technologies for effective policy implementation. Expanding access to extension officers could enhance farmers’ adaptive capacity and warrant the efficiency of implemented practices.

## 1. Introduction

In rural areas, agriculture is the principal source of income for around 70% of the world’s poor. The sector provides jobs for roughly 1.3 billion smallholders [1]. In 2050, there will be 9.1 billion people on the planet, and by the end of the century, there will be over 10 billion, and agriculture is required to meet this projected worldwide population growth [2,3]. Other related studies predict a warmer earth, with an average temperature increase of 0.2 °C in the next 30 years. Agriculture and its associated activities are the primary sources of rising GHGs in the atmosphere [4,5]. Climate change is forcing agricultural systems to shift, according to Branca et al. [6], and smallholder farmers need more capacity and stability to cope. Despite the fact that climate change affects us all, its undetectable influence on agriculture is particularly dangerous for developing countries, particularly those in Asia and Africa, where temperatures are already higher, and sees slow development [7,8,9,10,11,12].

Changing temperature and precipitation patterns endanger agricultural development. Climate change is already affecting agriculture, according to Yildiz [13]. The changes mentioned above will have immediate and long-term impacts on global food security [14]. Crop failure, yield loss, or soil deterioration may have compounding impacts on the local economy, global commodity markets, and food security [15]. Climate change sensitivity in agriculture is a significant issue for sustainable global food systems [16]. Smallholder farmers are especially susceptible to climate change consequences due to their small farm sizes, lack of funds, and restricted market access. Extreme system shocks, such as climate change, tend to hit the poorest and most vulnerable [17]. Smallholder farmers, especially those in developing countries, are most vulnerable to the effects of climate change, according to Anuga et al. [18]. However, agricultural systems are becoming increasingly sensitive to climate change, demanding adaptation, and changes to current agricultural processes. For example, the Food and Agriculture Organization (FAO) has developed the concept of climate-smart agriculture (CSA), which promotes farm-level innovations and sustainable practices. CSA is a set of actions that may increase farm resource efficiency while minimizing greenhouse gas emissions. CSA uses the current understanding of sustainable agricultural development to identify viable options and crucial supporting measures. Ecosystem services increase productivity, climate change adaptation, and mitigation initiatives.

Environmentally friendly farming practices, such as CSA, have been shown to reduce greenhouse gas emissions by up to 20% in only one year [19]. By reducing greenhouse gas emissions, CSAs can enable agricultural systems to adapt to climate change, according to Cramer [20]. It was found by Wambugu et al. [21] and Gikunda et al. [22] that creating awareness and building capacity are the only ways to ensure successful CSA adoption. Practices are spread and adopted in the context of social and cultural systems [23]. There is an increasing interest in climate-smart agriculture (CSA), particularly in developing countries, due to its promise to increased food security, climate change resistance, and greenhouse gas emission reduction [24]. As the agricultural industry in Africa is susceptible to climate change, climate-smart agriculture is essential for Africa’s future economic success [25]. When CSA technology and practices are used, the effects of climate change on agriculture may be lessened. Mainstreaming climate-smart agriculture (CSA) into the agricultural system has improved output and decreased greenhouse gas emissions [26,27,28]. The literature has widely accepted that the main reason for the low adoption rate of CSA practices and technologies, particularly for small landholding and marginalized farmers, is the limited access to financial resources [29,30,31,32,33,34].

Nelson et al. [35] asserted that climate change has increased the risks faced by farmers in developing countries. Although various measures have been implemented to minimize the effects of climate change on agriculture, the cost of these interventions is still high, despite the introduction of new farming techniques that can improve the efficiency of their operations [36]. Climate risks are still present in developing countries and, in most cases, affect the operations of rural farmers [37]. They cannot absorb the losses they incurred during the harvesting season.

Mensah et al. [38] recognized that the unavoidable threat that climate change poses to Ghana’s agriculture can disrupt the rural source of income through increased temperature levels, moisture deficits, land degradation, water shortages, increased incidence of alien diseases and parasites, and synergistic adverse effects on agriculture. Due to climate change, poverty reduction and food security could be threatened. It also makes agriculture and forestry specifically vulnerable. Consequently, it weakens the ability of the country. To achieve its sustainable development goals one and eight, Ghana has sought to end poverty in all its forms (particularly those who reside on less than USD 1.25 a day). It aims to encourage inclusive and sustainable economic development, employment, and practical benefit by 2030. Emanating from the issues outlined, the study seeks to ask whether the adoption of climate-smart agricultural practices (CSAPs) impact farmers’ income in Ghana.

A small but increasing body of research has examined how CSA adoption affects Ghanaian farmers’ incomes. However, the majority of these studies [6,18,33,38] did not investigate CSA practices’ impact on farmers’ income in Ghana. As a result, additional proof is needed to support how CSA adoption affect farmers’ incomes in Ghana. The impact of CSA adoption by farm households and the influence on farmers’ income examined in this paper will benefit the public, since the findings may shift local climate policy implementation towards sustainable agricultural growth and poverty reduction. The following is an outline of the rest of the essay. In Section 2, we lay out our theoretical foundation for the research project. To address the study’s research question, Section 3 covers the study area, survey design, and methodology. Results and discussion are presented in Section 4; the conclusion and implications of the research findings are presented in Section 5.

## 2. Incorporation of Climate-Smart Agriculture in Ghana and Empirical Review

A growing corpus of literature has emphasized the relevance of CSA adoption across the world [33,39,40]. The mainstreaming of climate change in Ghana has been slow since the Growth and Poverty Reduction Strategy II (2006–2009) [39]. As a result, the Ghanaian government has tried to include climate change into all aspects of development planning, particularly local development plans (known as Medium-Term Development Plans). “Deepening the mainstreaming of climate change in national and sub-national development planning and budgeting processes” and “promoting and documenting improved climate-smart indigenous agricultural knowledge” are among the goals of a new national development policy framework, An Agenda for Jobs: Creating Prosperity and Equal Opportunity for All (First Step) 2018–2021 [40]. When it comes to comprehending agricultural systems, it is essential to look at the whole picture, not just the individual components. Farming’s environment is shaped by interactions between public and private sectors, political factors, market infrastructure, and other institutional features [41,42,43].

Climate change and agriculture are well known in Ghana. According to the EPA, agricultural, forestry, and other land-use sectors (AFOLUs) account for 54.4% of Ghana’s greenhouse gas emissions [44]. Crop and animal pests and illnesses and salinization of agricultural soils owing to sea-level rise and tidal floods are already affecting farming in Ghana. The National Climate Change Committee (NCCC) was established in 2010, and released in 2010 and 2014, respectively, the National Climate Change Policy Action Program for Implementation in 2015 [39,45]. Studies have shown that CSA techniques increase the efficiency of resources and agricultural production and quadruple farmers’ incomes [46,47,48]. A World Bank team helped Ghana’s Ministry of Agriculture design a climate-smart agriculture investment plan. This paper outlines the World Bank and Ministry of Food and Agriculture 2020’s CSA policy and its implementation in agriculture. This paper outlines how these strategies would be executed considering Ghana’s diverse agroecological zones. The Ministry of Agriculture (MoFA) and development partners, World Bank, and FAO, promote CSA. CSA actors and technology have already been extensively deployed in numerous agricultural places worldwide [45].

On the other hand, Ali [49] explored how farm households perceive climate change, the factors that influence the adoption of climate-smart activities, and their relationship. Data from 704 farm households in Northern Togo were used to perform zero-inflated Poisson regression and multivariate analysis. The number of implemented solutions was determined mainly by household minimum consumption demands, gender, land availability, financing, and extension service. The most widely accepted agricultural strategies include the adoption of resistant and high-yielding cultivars, crop, and animal integration, soil and water conservation, organic fertilizer usage, and adjusting sowing time. The implemented agricultural development techniques were found to be highly complementary. Gender, household location, education level, family size, and labor allocation impact families’ adaptation strategy choices. Environmentally friendly activities may be promoted via institutional variables such as market access, finance availability, and extension services.

Sardar et al. [50] attempted to answer the same issue as to whether the adoption of climate-smart agriculture (CSA) strategies boosts farmers’ crop revenue. They conducted a study of 420 farmers in Punjab, Pakistan, in three different agroecological zones. Multinomial logistic regression was used to analyze the variables that influence the adoption of a single practice to a complete package of CSA practices in the research. It also employed a two-stage least squares estimation approach to manage the endogeneity issue and evaluate its conditional influence on crop output and farm revenue. These findings show that farmers who followed a comprehensive set of CSA practices had a 32 percent and 44 percent increase in production and a 45 percent and 48 percent increase in farm revenue compared to farmers who did not embrace the practices. In addition, the effect of adaptability differs depending on the level of CSA techniques used by the farmers.

Ighodaro et al. [51] examined the impact of CSA practices on smallholder farmers’ total income, using the Qamata Irrigation Scheme, South Africa, as an example. Data were gathered from the scheme’s seventy smallholder farmers using a case study research approach, and analysis was done using a multiple linear regression model, since the dependent variable was continuous. According to the findings, older farmers were more likely to see a rise in total income. It was more probable that farmers who were aware of best practices would improve their total revenue. Female-headed families and farmers who have a negative view of extension advice were both shown to have a greater likelihood of increasing total income, which was unexpected. Soil conservation efforts by smallholder farmers at Qamata Irrigation Scheme have a considerable impact on farmers’ total income in the studied region.

Gikunda et al. [22] argued that climate change harms food and animal feed production. According to research, greenhouse gas emissions are the principal cause of climate change [52]. Climate-smart agriculture (CSA) may offset GHGs such as CO_2_ by up to 20% in a year [19]. CSA helps agricultural systems adapt to climate change by reducing GHG emissions. According to Wambugu et al. [53], CSA implementation requires raising awareness and improving capability. Practices spread and are adopted in social and cultural systems [23]. Language, traditions, agricultural methods, heredity, conventions, values, religious beliefs, politics, social structures, attitudes, and taboos are all part of culture [54]. Farmers’ attitudes, goals, and expectations are also influenced by the society’s culture and social groupings, where they serve as members. Notably, farmer attitudes influence technology uptake [19]. Despite the rising volume of research on CSA [18], little is known about CSA adoption’s factors. Research shows how social structures impact innovation spread and acceptance [23].

### Conceptual Framework of CSA

**Figure 1:**
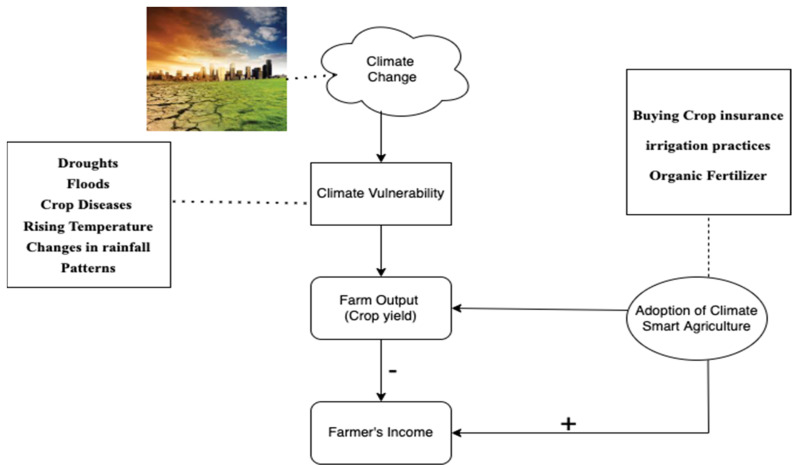
Conceptual framework on climate change, its repercussions, climate-smart agriculture practices and farmers’ income.

Almost three-quarters of the world’s poor live in rural regions, and agriculture is the primary source of their income. In the absence of mitigation efforts, agriculture will bear the brunt of climate change’s negative impacts. Agriculture, for example, was responsible for 26% of all damage caused by climate-related catastrophes in emerging nations between 2006 and 2016 [55,56]. As portrayed in Figure 1 below, climate change leads to climate vulnerabilities, including droughts, floods, rising temperature, and changes in rainfall patterns, which negatively influence farm productivity. With no action on climate change, many rural farmers would lose their livelihoods because of the consequences of climate change on agriculture [56]. Indirectly, climate change reduces agriculture yields, which lowers farmers’ income. Results from climate change impact models released by South Africa’s Financial and Fiscal Commission (FFC) in 2015 show that agricultural yields are the most severely affected by global warming. As a result, millions of Africans who rely on agriculture for their food and income have been cautioned by Schulze [57] that climate change would influence agricultural output and create risks linked with pauses in production. The adoption of climate-smart agricultural practices such as buying crop insurance, irrigation practices, and organic fertilizer could aid in the mitigation of climate change on agriculture. Climate-smart agricultural methods can help farmers double their income, which is one of the many benefits of adopting these strategies. The primary goal of CSA is to boost productivity and raise income [55,58].

## 3. Methodology

### 3.1. Description of the Study Areas

**Figure 2:**
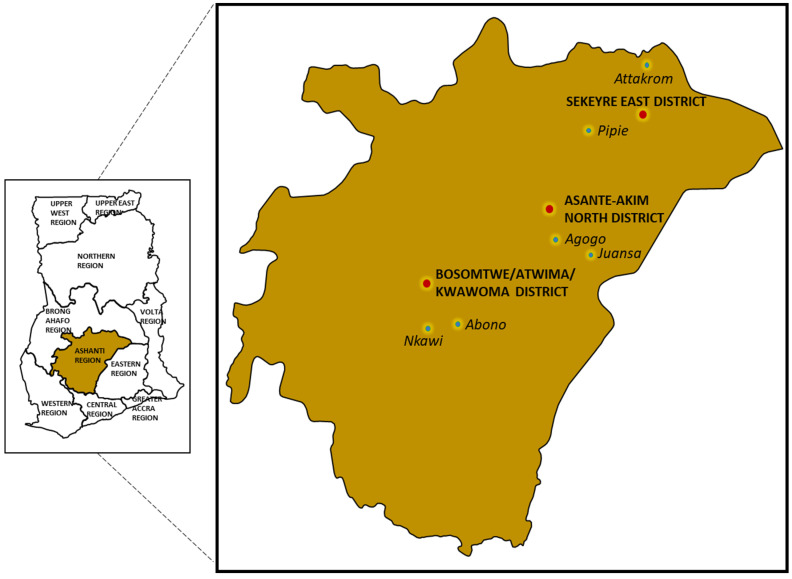
Map showing study districts and selected villages.

The study utilized a cross-sectional design to analyze the characteristics of interest. This method helps in uncovering the various factors that influence cocoa farmer’s decisions. We present detailed information on the data collected, study area, and the model specification under this section. There were 10 regions in Ghana, namely Ashanti, Brong-Ahafo, Central, Eastern, Greater Accra, Northern, Upper East, Upper West, Volta, and Western region. As for the new regions, they were added in 2018. This study only considered the existing regions since they do not have up-to-date population details. The Ashanti Region has a population of approximately 4,780,380. Out of this, 2,316,052 are males and 2,464,328 are females. It is in the middle belt of the country. The Ashanti Region is divided into three regions: Central, Western, and Southern. According to studies conducted by the Ministry of Food and Agriculture, most of the region’s residents are engaged in agriculture. The activities related to farming and fishing are also conducted in the region. Figure 2 presents the map of Ashanti Region having dotted red colour as the districts and dotted blue colour as the villages for the study area.

### 3.2. Site and Sample Selection

This study includes all the cocoa farmers in the selected districts in the Ashanti Region. The population, including those who live in the 6 cocoa production villages, were conveniently selected out of the 3 districts, which include Nkowii, Pipie, Attakrom, Abono, Agogo, and Juasan. The multistage sampling procedure was considered based on the dominants of literature. This study aims to introduce the concept of the purposive sampling method for selecting the Ashanti Region, since it is one of the most productive regions in the country. The second part of the study involved the selection of 3 major districts in which cocoa production is a flourishing economic activity. This was done using a random sample size of 600 farmers. The researchers interviewed eight farmers to learn about their socioeconomic and demographic features, agricultural risks, and revenue sources. The effectiveness of various agriculture insurance policies and farming practices was also evaluated. The pre-test was intended to reduce unnecessary questions and improve interview quality. Based on the pre-test survey results, the study components were revised. The project also employed three graduates to help interview cocoa producers. They were taught how to ask the correct questions to the farmers. We preferred to interview at least 10 to 15 people per day because returning for follow-up interviews is difficult. This strategy worked for 600 questionnaires. The sample size was calculated using the sample size formula provided by Godden (2004) for an infinite population greater than 50,000. From Table 1, it is evidenced that the total population is more significant than 50,000, hence making the formula suitable for the calculation of the sample for the study. The formula is explained below and how the sample size has been arrived at.

$$\mathrm{SS}=\frac{Z^{2}\times p\times \left(1-p\right)}{M^{2}}$$

where, SS = sample size; Z = Z-value (e.g., 1.96 for 95% confidence level); p = percentage of population picking a choice, expressed in decimal; M = margin of error.

$$\mathrm{SS}=\frac{{\left(1.96\right)}^{2}\times 0.5\times \left(1-0.5\right)}{0.04^{2}}$$

$$\mathrm{SS}=\frac{3.8416\times 0.5\times 0.5}{0.0016}=600.25$$

Hence, the sample considered for the cocoa farmers in the selected districts in the Ashanti Region was 600.

### 3.3. Data and Variables

The study relied on both primary and secondary data. The data came from Cocoa farmers in the various districts within the region. The secondary data were gathered through various sources such as academic journals, textbooks, and reports. The questionnaire was designed to collect data on various aspects of farming and households. The questionnaire was divided into four main sections. The first section of the questionnaire was on household characteristics, followed by farm characteristics as the second section, and, finally, farmers’ income sources and income level as the third section. Table 2 below presents the description of variables and their measurements. Multi-collinearity occurs when the correlation between the independent variables in a regression model is high. When there is a high degree of correlation between variables, modeling and analysis might become difficult. Prior to data analysis, the contingency coefficient test was used to diagnose co-linearity and rule out independent variables significantly related to one another. Table 3 summarizes the findings. The low correlation coefficients between the independent variables demonstrate no multi-collinearity among the variables included in our study.

### 3.4. Analytical Framework

The study used an endogenous switching regression framework to examine the effects of climate-smart agricultural practices’ adoption on farmers’ income. The model was derived from the Heckman selection correction approach. It is most likely that the self-election of cocoa farmers triggered the selection bias. An endogenous switching regression model accounts for the selection bias that may have occurred due to self-selection of employed households [50,59,60]. It was assumed that every cocoa farmer has an equal probability of adopting a climate-smart agricultural practice. This means that, even though they have the same probability of adopting the policy, they could still choose not to adopt it. The selection bias problem in impact evaluation is a problem that can be solved using the endogenous switch regression model. This procedure is usually performed in two phases: first, it estimates the actual outcomes, and second, it accounts for the unobserved heterogeneity. A factor model can be used in the second stage to estimate the non-adopters of CSA. The method is based on the ESR model. It avoids the need for endogeneity validation using selectivity. The ESR model can also account for the unobserved variables. This feature allows the group to be monitored, allowing the answers to be different. We estimated the selection equation as follows:

(1) $$\lambda_{i}^{*}=\delta +\varphi \beta_{i}+\varepsilon_{i}$$

where $\lambda_{i}^{*}$ takes the value of 1 if a cocoa farmer adopts CSA and 0 otherwise. δ represents the intercept and $\beta_{i}$ denotes a vector of exogenous variables that affect the decision to adopt CSA; φ signifies a vector of coefficient and $\varepsilon_{i}$ is the error term. The second phase of the endogenous switching model used a full information maximum probability (FML) model to account for selection bias. The model represented various switching regimes with binary outcomes. The switching regimes are expressed as:

(2) $$\mathrm{Regime}\;1:\;\Gamma_{1i}=k_{1i}\vartheta_{1}+\rho_{1\varepsilon }\gamma_{1i}+\mu_{1i}\;\mathrm{if}\;R_{i}=1\;\mathrm{for}\;\mathrm{adopters}\;\mathrm{of}\;\mathrm{CSA}$$

(3) $$\mathrm{Regime}\;2:\;\Gamma_{2i}=k_{2i}\vartheta_{2}+\rho_{2\varepsilon }\gamma_{2i}+\mu_{2i}\;\mathrm{if}\;R_{i}=0\;\mathrm{for}\;\mathrm{non}\text{-}\mathrm{adopters}\;\mathrm{of}\;\mathrm{CSA}$$

$\Gamma_{i}$ in Equations (2) and (3) denotes the outcome variables of cocoa farmer i for each regime. $k_{i}$, on the other hand, represents a vector of factors that determine CSA adoption. The variable in vectors k in Equations (2) and (3) may overlap with the β in Equation (1) if the latter does not appear in β. To estimate the income estimation formula, the two vectors must have at least one variable that does not appear in β. ϑ and ρ were parameters to be estimated, and $\mu_{1i}$ and $\mu_{2i}$ were independently and identically distributed error terms. The Inverse Mills Ratio is a statistical representation of participation in a selection process. It is used to correct for selection bias in two-step estimation procedures. It is expressed as:

$$\gamma_{1i}=\frac{\theta \left(\lambda_{i}\delta \right)}{\phi \left(\lambda_{i}\delta \right)}\;\mathrm{and}\;\gamma_{2i}=\frac{\theta \left(\lambda_{i}\delta \right)}{1-\phi \left(\lambda_{i}\delta \right)}$$

The null hypothesis that the absence of a selection bias is not caused by selection bias would be rejected. The non-zero covariance between the outcome equation and the error terms of the selection procedure would be rejected. The covariance of the error terms $\Gamma_{1i}$ and $\Gamma_{2i}$ is undefined since they cannot be observed simultaneously. However, through the $\mu_{1i}-\mu_{2i}\;$correlation, the existence of the trivariate normal distribution is assumed. This is defined as:

(4) $$cov\left(\mu ,\mu_{1},\mu_{2}\right)=\left[\begin{array}{ccc} \varphi_{1}^{2} & \varphi_{1}\varphi_{2} & \varsigma_{1z}\varphi_{1}\\ \varphi_{1}\varphi_{2} & \varphi_{2}^{2} & \varsigma_{2z}\varphi_{1}\\ \varsigma_{1z}\varphi_{1} & \varsigma_{2z}\varphi_{2} & \varphi_{\mu }^{2} \end{array}\right]$$

In Equation (4), the covariance between the disturbance terms of the outcome equation and selection model was presented as cov(ε,μ)=ς. Thus, $\varsigma_{1z}$ and $\varsigma_{2z}$ were the correlation coefficients between $\mu_{1i}$ and $\varepsilon_{i}$ and between $\mu_{2i}$ and $\varepsilon_{i}$, respectively.

Our main objective was to estimate the average treatment effects and the change in the incomes of cocoa farmers due to the adoption of CSA practices, estimated as the difference between adopters and non-adopters. The complexity of the two treatment-effect assumptions makes model selection critical in estimating treatment effects. When relying on a set of control variables, the conditional independence assumption is more likely to be met. The overlap assumption assumes that the probability that a given unit will be treated is always positive. It is also more confident when the model contains fewer variables. This paper has several control variables, and, therefore, considered a model selection method that can identify all the covariates in the model. We followed Koch et al. [61] and Leeb et al. [62] and employed the telasso method, which can help avoid selecting the wrong covariates in the model. While estimating treatment effects, telasso uses lasso techniques to select the appropriate variable sets. The teffects AIPW command is similar to the AIPW command in that it estimates the effects of only one outcome or treatment model. Telasso is an AIPW estimator that allows for selecting covariates through lasso. It is robust against functional and non-factual form misspecification [63]. In this paper, we propose that the model should have many possible constraints and that lasso should be able to select from among them, as recommended by Farrell [64] and Chernozhukov et al. [63]. We denote the average treatment effect by $\Gamma_{i}$ (farm income, household income, and off-farm income), as portrayed in the following equation. They are defined as:

(5) $$\Gamma_{i}=\sigma_{0}\left(\varsigma_{i}\vartheta_{i}\right)+\mu_{i}$$

$$E\left(\mu_{i}|\varsigma_{i}\vartheta_{i}\right)=0$$

where $\Gamma_{i}$ is the outcome variable (farm income, household income, and off-farm income), $\varsigma_{i}$ is the binary treatment variable; thus, for farmers who adopt and do not adopt the climate-smart agricultural practices, $\vartheta_{i}$ denotes the control variables in the model, and $\sigma_{0}\left(\varsigma_{i}\vartheta_{i}\right)$ is the expected potential outcome given a level of treatment and covariates $\vartheta_{i}$. Because each technology can only be treated or not treated, the observed outcome $\Gamma_{i}$ can only be of $\sigma_{0}\left(1,\vartheta_{i}\right)+\mu_{i}$ or $\sigma_{0}\left(0,\vartheta_{i}\right)+\mu_{i}$.

The treatment effect model of the lasso model takes the following form:

(6) $$\varsigma_{i}=\gamma_{0}\left(\lambda \right)+\mu_{i}$$

(7) $$E\left(\mu_{i}|k\right)=0$$

where λ are the potentially high-dimensional covariates in the treatment model. $\gamma_{0}\left(\lambda \right)$ represents the expected value of $\varsigma_{i}$ given λ. Thus, $\gamma_{0}\left(\lambda \right)$ is the probability of a farmer being treated, given λ. Our parameter of interest in this paper is the average treatment effect (ATE) and average treatment effect on the treated (ATET), which are expressed in the equations below:

(8) $$\mathrm{ATE}\;\mathrm{is}\;\varphi_{0}=E\left\{\sigma_{0}\left(1,\vartheta_{i}\right)-\sigma_{0}\left(0,\vartheta_{i}\right)\right\}$$

(9) $$\mathrm{ATET}\;\mathrm{is}\;\varphi_{0}=E\left\{\sigma_{0}\left(1,\vartheta_{i}\right)-\sigma_{0}\left(0,\vartheta_{i}\right)|\;\varsigma_{i}=1\right\}$$

## 4. Results and Discussion

### 4.1. Descriptive Results

We present the mean differences between the variables adopted for the study in the characteristics of climate-smart agricultural practices. Table 4 revealed that education, asset ownership, household size, access to credit, awareness of CSA, visitation of extension officers, and risk-averse had a significant difference between cocoa farmers who adopt and do not adopt irrigation on their farms. The positive coefficient of education, asset ownership, and risk-averse implies that farmers who adopt irrigation as a climate-smart practice are highly educated, have more assets, are aware of CSA practices, and are risk-takers as compared to non-irrigators. However, irrigators obtain significantly smaller household sizes and access to credit than non-irrigators. Vis-à-vis outcome variables, we demonstrate no significant mean disparity in household and off-farm income between irrigators and non-irrigators. The mean level of farm income for farmers who adopt irrigation as a CSA practice is significantly higher than farmers who do not practice irrigation.

Table 4 further portrayed that gender, asset ownership, access to credit, awareness of CSA, and visitation of extension officers had a significant difference between cocoa farmers for adopters of crop insurance and non-adopters of crop insurance. We find out that adopters have lower assets and lower access to credit than their counterparts. Similarly, farm and off-farm outcome variables exhibited a lower average income for adopters of crop insurance than non-adopters. Finally, regarding our third climate-smart practice (adoption of organic fertilizer), farm experience, household size, and farm labor are statistically significant in their mean differences. Under this climate-smart practice, we deduce that adopters of organic fertilizer had a significantly lower farm income as compared to non-adopters of organic fertilizer.

In any case, the mean assessments do not consider confounding dynamics that may skew the effects of irrigation, crop insurance, and organic fertilizer adoption on farmers’ income levels. The data in Table 4 cannot be used to conclude the effects of the climate-smart agricultural practices employed in this study on farmers’ incomes. Moreover, all control variables differ considerably from the climate-smart practices used from each other in Table 4. We therefore utilized the ESR model as a robust econometric technique to address the unbiased effects of irrigation, crop insurance, and organic fertilizer adoption on farmers’ income, following the works of Li et al. [65] and Hou et al. [66].

**Figure 3:**
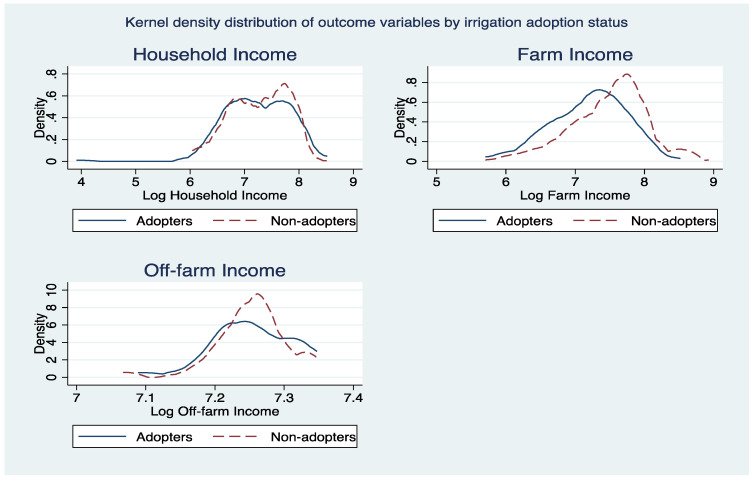
Kernel density distribution of outcome variables by irrigation adoption.

**Figure 4:**
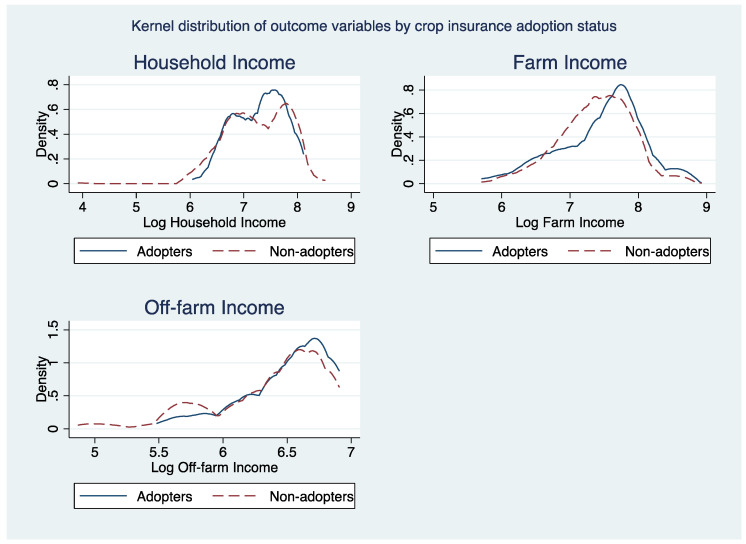
Kernel density distribution of outcome variables by crop insurance adoption.

**Figure 5:**
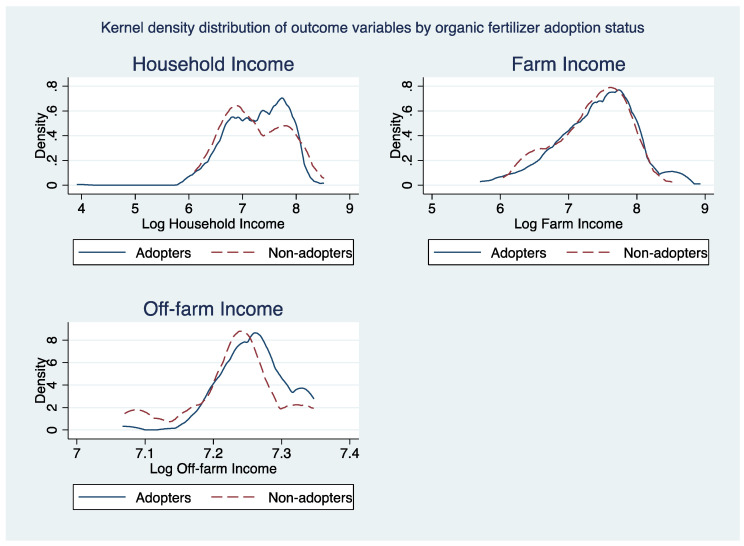
Kernel density distribution of outcome variables by organic fertilizer adoption.

Figure 3, Figure 4 and Figure 5 portrays kernel density distributions of household, farm, and off-farm income by the adoption status of the cocoa farmers. The distributions of these data highlight the significant differences between the different climate-smart agricultural practices of non-adopters and adopters. Thus, the ESR model has been proven to be correct for the study.

### 4.2. Determinants of Climate-Smart Agricultural Practices

Table 5 below displays the determinants influencing irrigation, crop insurance, and organic fertilizer adoption. Due to the complexity of the statistical data, we analyzed the marginal effects of the various explanatory variables. The result indicates that gender, farm experience, age, household size, and farm size do not significantly influence the adoption of irrigation and crop insurance in our study. This outcome c66an be attributed to several reasons, including population characteristics, confounds, or outside factors. The result is consistent with the findings of Li et al. [65]. Gender influences the adoption of organic fertilizer positively and statistically significant at a 10 percent level. This implies that female household heads are more likely to adopt organic fertilizer than their male counterparts. This finding contradicts the findings of Foudi and Erdlenbruch [67] and Li et al. [65], wherein they argued that women tend to receive less off-farm work than male household heads, which means they are more likely to engage in farm management activities.

Farm experience had a negative impact on the adoption of organic fertilizer. An extra experience gained in farming leads to a 19.9% decline in the adoption of organic fertilizer, all things being equal. The size of a household is positively related to the adoption of organic fertilizer. Similar results were found in the works of Chikowo et al. [68] and Daadi and Latacz-Lohmann [69], in which they explained that the positive coefficient of household size is due to the labor-intensive use of organic fertilizer and, therefore, households with the capacity to meet the requirements will be more likely to adopt the practice. As evidenced in our findings, increases in farm size are statistically linked to a 0.13% decrease in the adoption of organic fertilizer. This finding is consistent with Daadi and Latacz-Lohmann’s [69] study. Farm association membership increases farmers’ likelihood of adopting organic fertilizer, irrigation, and crop insurance. A possible reason for this could be improved access to information and knowledge sharing among farmers in these associations. These findings are in line with previous studies [69,70,71,72].

Asset ownership is positively and significantly associated with the adoption of crop insurance. Crop insurance is a novel technique that farmers are likely to adopt with a more significant farm asset. The likelihood of using crop insurance increased by 0.30% for resource-rich farmers. Crop insurance as a climate-smart agricultural practice is likely to be more accessible to farmers with more assets than those with fewer assets. It is argued that these assets help absorb the risks of failure and the amount of time it takes for CSAP to have a substantial impact. It is also worth noting that wealthier people can afford to invest in these initiatives to boost crop yields. According to Wekesa et al. [73], Teklewold et al. [74], and Ochieng et al. [75], the lack of productive assets limits the ability to embrace climate-smart practices that necessitate enormous resource allocations. Our findings are in line with their findings, as are those of the aforementioned authors. A rise in farm assets may diminish the likelihood of using irrigation as a CSAP.

Our result portrayed a significant positive impact of access to credit on the adoption of irrigation and crop insurance but a negative effect on the use of organic fertilizer. The results suggest that farmers who received credit were 0.41% and 0.45% more likely to adopt irrigation and crop insurance, respectively. The ability to obtain credit helps farmers reduce their expenses related to implementing CSAP technologies. However, it can also affect the uptake of these products by households less likely to have cash outlays. The finding is consistent with Wekesa et al. [73] and Ali [49]. Finally, irrigation and crop insurance uptake are negatively and statistically significant with limited access to extension services. A lack of access to extension officers can influence the adoption of new technologies. Farmers may opt to employ traditional means if they have limited access to extension services due to global warming to reduce the risk of crop failure. Farm membership significantly increases the probability of irrigation and crop insurance adoption. A unit increase in farm membership leads to a 38.5% and 77.4% increase in the adoption of irrigation and crop insurance, all things being equal. Obtaining the necessary training and support from other farmers can help boost the likelihood of crop insurance and irrigation adoption. The findings are consistent with previous studies [33,69,70].

### 4.3. Determinants of Cocoa Farmers Income

Factors affecting farm income, household income, and off-farm income are shown in Table 6, Table 7 and Table 8, respectively, using the estimations from Equations (2) and (3). The results support the use of the ESR model to estimate the effects of CSAP.

Table 6, Table 7 and Table 8 present the reports on the adoption of irrigation, crop insurance, and organic fertilizer and their impact on farmers’ income. With household income as the outcome variable, farm experience had a negative impact on household income for non-adopters of irrigation as a CSAP. For non-adopters with off-farm income, farm experience is positive and statistically significant. Farm experience positively impacted household income for non-adopters of crop insurance and the use of organic fertilizer as a CSAP. For adopters of crop insurance and organic fertilizer, farm experience positively impacted their farm and off-farm income, respectively. The results are statistically significant at a 10% significant level. Aside from using new equipment and services, highly experienced farmers also require more agricultural inputs to enhance their operations. Thus, a 1% percent increase in farm experience will influence farm and off-farm income, respectively. Farmers who adopt irrigation practices do not need to wait for running season to plant crops and therefore have ample time to engage in off-farm activities to generate more income. A possible explanation for this is that experience will educate farmers on efficiently utilizing climate-smart practices that can save time, resources, and energy. For instance, Deressa et al. [76] and Ali [49] posited that education increases farmers’ adaptive capacities. Thus, the more farmers are educated, the more likely they are to adopt smart agricultural technologies, thereby increasing their productivity. Therefore, there is a significant negative coefficient output of education on adopters and non-adopters of crop insurance, as this CSAP implies that having lower education decreased the probability of doubling non-adopters’ household and farm income by roughly 9.6% and 10.6%, respectively. However, education had a significant positive effect on the household and off-farm income of adopters of organic fertilizer.

For adopters of irrigation, asset ownership (farm machinery in this study) has a negative and significant impact on household income and farm income. The result is statistically significant at a 10% significance level, respectively. As essential production equipment, farm machinery improves farmers’ efficiency when undertaking farm activities, and a lack of it will drastically affect farm productivity. Asset ownership negatively influences adopters of crop insurance as a CSAP. The outcome is consistent with the result of adopters of irrigation as a CSAP. Asset ownership also increases farm income for non-adopters of irrigation and crop insurance as a CSAP. This result affirms the earlier assertion and corroborates the findings of Ma and Wang [77] and Ma and Zheng [78]. On the other hand, asset ownership doubles household and farm income for adopters of organic fertilizer as a CSAP, implying that farmers who adopt organic fertilizer turn to increase their household and farm income more than their counterparts.

Household size positively influences the impact of the income level of farmers. The household size also affects the amount of household income, farm income, and off-farm income for climate-smart practices such as irrigation and crop insurance. This study shows that, as the number of dependents rises, household income, farm income, and off-farm income increases. The findings are consistent with Ghimiré et al. [79] and Kehinde [80]. However, for climate-smart practices such as organic fertilizer, a large household size negatively influences farm and off-farm income. However, farm size had a mixed influence based on the income level. For both adopters and non-adopters of irrigation and organic fertilizer on household income and off-farm income, farm size has a positive and significant impact, proposing that cocoa farmers with larger farms tend to earn a higher household and off-farm income. This finding is congruent with Li et al. [65]. Moreover, household size has a significant negative impact on farm income for adopters of irrigation as a CSAP. A possible explanation could be that large farms tend to encourage more production and are capital intensive in management. The result agrees with Kehinde [80], Ma and Wang [77], and Meraner et al. [81].

The coefficient of access to credit is positive for all income levels except the farm income of non-adopters, suggesting the vital role of access to credit in farming. Moreover, access to credit revealed a significant positive impact on household income for adopters and non-adopters of organic fertilizer. Non-adopters maintain a consistently positive impact of access to credit on-farm and off-farm income, whereas adopters portrayed a negative relation with farm and off-farm income. Since credit is a vital component of cocoa farming in Ghana, it can help ease the financial constraints faced by the farmers. Cocoa farmers with access to credit are better positioned to overcome financial constraints and increase their income levels compared to their counterparts. The finding is consistent with the results of Kehinde [80], Owusu et al. [82], and Yeboah [83].

Extension officers, among other things, aid farmers in adopting current agricultural technology and methods. The results of access to extension officers positively impact farmers’ income for adopters of irrigation as a CSAP. Moreover, access to extension officers renders a positive, statistically significant effect on household and farm income for adopters of crop insurance as a CSAP. However, the opposite effect is portrayed for non-adopters of crop insurance.

The findings show that access to extension services would enhance the amount of irrigation adaption and crop insurance. This implies that a unit increase in irrigation and crop insurance adoption would increase farm income by 10.4%, 10.3%, and 20.3%, all things being equal. The findings align with Ali [49] and Deressa et al. [84]. Non-adopters who lack the connection with extension officers to be enlightened on innovative agricultural practices could earn a lower income. As expected, the instrumental variable (farm association membership) had a statistically significant impact on all the climate-smart agricultural practices adopted in the study. Farm association membership increases farmers’ likelihood of organic fertilizer, irrigation, and crop insurance adoption in the study area. This is due to the improved access to information and knowledge sharing among farmers in these associations. The findings are in line with previous studies [69,70,71,72].

### 4.4. Impact of Climate-Smart Agricultural Practices on Farmers’ Income

Table 9 below presents the empirical analysis on the impact of climate-smart agricultural practices on farmers’ income. In estimating the average treatment effect, we employed the telasso technique, which permits the outcome variable to be modeled using linear, logistic, probit, or Poisson model due to its robustness to model misspecification in either the outcome or the treatment model. We further performed a robust analysis using nearest neighbor matching and propensity score matching average treatment effects (refer to Table 10). Results from Table 9 reveal that the adoption of climate-smart agricultural practices has a positive impact on the income level of farmers. All climate-smart practices adopted for the study have a positive coefficient, suggesting that a unit increase in the adoption of climate-smart agricultural practices will influence farmers’ income positively. For instance, adopter’s percentage, if all respondents were to adopt irrigation, is 8.6 percentage points higher than the average of 65 percent that would be expected if all cocoa farmers were not to adopt irrigation. This implies that cocoa farmers who adopt irrigation will earn 8.6% and 11.1% more on their total household income and farm income than their counterparts. The finding is in line with the results of Li et al. [85], Burney and Naylor [86], and Dillon [87], where the findings of these studies show that access to irrigation has a significant impact on rural incomes.

With the adoption of crop insurance, the coefficient of average treatment effect has a positive and statistically significant on household, farm, and off-farm income, whereas with irrigation, only household and farm income are statistically significant. On the other hand, organic fertilizer also positively impacts farmers’ income but is statistically insignificant on all the income levels. This implies that, if all cocoa farmers were to adopt crop insurance, they would be expected to earn 15.9, 14.2, and 17.9 percentage points more than the average of 63.2, 73.2, and 62.3 percent of their household, farm, and off-farm income. By implication, cocoa farmers who adopt crop insurance for their cocoa farms would increase their household, farm, and off-farm income with 16%, 14%, and 18%, respectively. This paper concludes that climate-smart practices positively influence farmers’ income in Ghana. The results are consistent with previous studies, including Ma and Zheng [78], Li et al. [65], Tong et al. [88], Zhao et al. [89]. We conjecture that, among irrigation, crop insurance, and organic fertilizer as climate-smart agricultural practices, crop insurance is the most efficient and effective climate-smart practice, since it has the highest positive coefficient and is statistically significant on all incomes.

### 4.5. Robust Analysis on the Impact of Climate-Smart Agricultural Practices on Doubling Farmers’ Income

We further employed two matching algorithms, namely, nearest neighbor (NNM) and propensity score matching (PSM) techniques, to compare with the telasso technique employed in the study. A matching study is an essential step in estimating the treatment effect. It involves estimating the covariate distribution of the treated and control groups. This study uses propensity score matching and nearest-neighbor matching to investigate the robustness of the results presented in Table 9. Table 10 below presents the results of NNM and PSM estimations. The results are similar, with a slight difference in magnitude and statistical significance to the telasso output. Thus, in using the NNM and PSM techniques, irrigation, crop insurance, and organic fertilizer maintain positive signs. Irrigation and crop insurance are still statistically significant on cocoa farmers’ household, farm, and off-farm income. By implication, the outcome portrayed in Table 8 highlights that NNM and PSM techniques are consistent with the telasso treatment effect.

## 5. Conclusions

Millions of African smallholder farmers are already feeling the effects of climate change. As a result, their crops have produced less, and their communities’ food security has been compromised. Due to the various effects of climate change on various aspects of agriculture, adaptation measures are needed to build the capacity of smallholders to manage their risks. This paper focuses on West Africa’s semi-arid regions. Because climate-smart agriculture techniques are still a relatively new notion, farmers may have only had partial exposure to them. Non-exposure bias affects adoption rates when a new technology is introduced to a target group that has not been fully exposed. The estimated adoption rate for various CSA practices and technologies is distorted due to the non-exposure bias. A consistent approach should be used to determine the adoption rate. Emanating from the issues above, this current study employed an endogenous switching regression coupled with a telasso average treatment effect to examine the impacts of climate-smart agricultural practices’ adoption on farmers’ income. We found that gender, farm experience, age, household size, and farm size do not significantly influence the adoption of irrigation and crop insurance in our study. Moreover, the study concludes that farm experience had a negative impact on the adoption of organic fertilizer, whereas the size of households positively influences the adoption of organic fertilizer. Farm association membership also increases farmers’ likelihood of adopting organic fertilizer, irrigation, and crop insurance. Our results revealed a significant positive impact of access to credit on adopting irrigation and crop insurance.

On the determinants of factors that influence climate-smart practices on farmers’ income, we found that farm experience is statistically significant for non-adopters with off-farm income. Non-adopters of crop insurance and organic fertilizer as CSAPs benefited from farm experience. Farm experience positively increases farm and off-farm income for crop insurance and organic fertilizer adopters. In contrast, asset ownership had a negative impact on household and farm income. We further revealed that household size also affects the amount of household income, farm income, and off-farm income for climate-smart practices such as irrigation and crop insurance. However, for smart climate practices such as organic fertilizer, a large household size negatively influences farm and off-farm income. Farm size had a mixed influence based on the income level. On access to credit, a positive coefficient was found for all income levels except the farm income of non-adopters. Moreover, access to extension officers renders a positive, statistically significant effect on household and farm income for adopters of crop insurance. However, the opposite effect is portrayed for non-adopters of crop insurance.

The adoption of climate-smart practices has a positive coefficient. This indicates that, if all the respondents in a given region adopted these practices, their income would increase significantly. This study shows that adopting irrigation practices leads to an increase in household income of 8.6% and 11.1%, respectively, for cocoa farmers. Crop insurance has a positive coefficient and is statistically significant for on-farm, off-farm, and household income. This paper shows that climate-smart practices, such as crop insurance, can positively influence farmers’ income in Ghana. We also conjecture that crop insurance is the most effective and efficient climate-smart practice among the various agricultural practices. The study suggests that access to credit and mass awareness should be made compulsory modules, coupled with the consistent training of farmers on new technologies for effective policy implementation. Expanding access to extension officers could enhance farmers’ adaptive capacity and warrant the efficiency of implemented practices. Finally, to improve farmers’ standard of living, climate-smart agricultural practices such as irrigation, crop insurance, and organic fertilizer adoption should be endorsed.

On limitations, the study was limited to the Ashanti Region of Ghana and specifically to cocoa farmers and not all farmers. This creates a gap in the literature that future researchers can address with innovation to the topic.

## Figures and Tables

**Table 1:** Sample Distribution.

Selected Districts	Population	Percentage	Proportion to Sample
Bosmtwe	93,910	35	210
Sekyere East	90,477	33	198
Akim North	87,501	32	192
Total	270,130	100	600

Source: Author’s calculation (2019).

**Table 2:** Variable names and measurements.

Variables	Measurement	Mean	S.D.	Observation
Gender	Binary variable = 1 (male), 0 (female)	1.388	0.488	600
Education	Continuous Variable = (in Years)	1.950	0.731	597
Farm experience	Continuous variable (in Years)	18.977	12.974	600
Household size	Continuous variable (number)	5.803	1.799	600
Age	Continuous variable (in Years)	43.872	6.805	600
Asset ownership	Binary variable 1 (yes), 0 otherwise	2.368	0.968	600
Farm size	Continuous variable (in acres)	6.622	9.008	593
Access to credit	Binary variable = 1 (have access), 0 otherwise	1.587	0.493	600
Extension officer	Binary variable 1 (yes), 0 (no)	2.135	1.034	600
Farm membership association	Binary variable = 1 (member), 0 otherwise	1.658	0.475	600
Climate-Smart Agricultural Practices variables				
Adoption of crop insurance	Binary variable = 1 (yes), 0 otherwise	0.327	0.469	600
Adoption of irrigation	Binary variable = 1 (yes), 0 otherwise	0.322	0.468	600
Adoption of organic fertilizer	Binary variable = 1 (yes), 0 otherwise	0.565	0.496	600
Outcome Variables				
Household income	Continuous variable (Ghc value)	7.262	0.554	534
Farm income	Continuous variable (Ghc value)	7.358	0.603	576
Off-farm income	Continuous variable (Ghc value)	6.372	0.773	529

Source: Field Survey, 2019.

**Table 3:** Contingency coefficient test for co-linearity between independent variables.

Variables	Gender	Education	Farm Experience	Age	Asset Ownership	Household Size	Farm Size	Access to Credit	Extension Officer	Farm Membership	Irrigation	Crop Insurance	Organic Fertilizer
Gender	1.000												
Education	0.039	1.000											
Farm Experience	0.058	−0.047	1.000										
Age	0.088	−0.052	0.007	1.000									
Asset Ownership	0.148	0.211	−0.021	0.105	1.000								
Household size	−0.059	0.017	0.042	−0.062	0.026	1.000							
Farm Size	0.055	0.022	0.060	0.049	−0.044	0.005	1.000						
Access to Credit	0.056	0.095	−0.023	−0.037	0.120	0.003	−0.030	1.000					
Extension officer	0.117	−0.031	0.025	0.049	0.034	−0.033	0.068	0.155	1.000				
Farm Membership	−0.016	−0.052	0.022	−0.057	0.079	−0.007	0.026	0.016	−0.033	1.000			
Irrigation	0.023	−0.015	0.061	−0.004	−0.061	0.076	0.005	0.099	0.189	−0.049	1.000		
Crop Insurance	0.068	0.107	−0.037	−0.010	0.133	0.014	−0.016	0.069	−0.110	−0.006	−0.038	1.000	
Organic Fertilizer	0.002	−0.022	−0.018	−0.019	0.011	0.070	−0.019	0.006	−0.014	0.074	−0.020	0.245	1.000

Source: Author’s computation based on survey data (2019).

**Table 4:** Mean differences between adopters and non-adopters of climate-smart agricultural practices.

Variables	Adoption of Irrigation		Buying of Crop Insurance				Adoption of Organic Fertilizer		
	Adopters	Non-Adopters	|t|	Adopters	Non-Adopters	|t|	Adopters	Non-Adopters	|t|
Gender	1.404 (0.492)	1.381 (0.486)	−0.55	1.439 (0.498)	1.364 (0.482)	−1.77 *	1.389 (0.488)	1.387 (0.488)	−0.06
Education	1.885 (0.709)	1.980 (0.740)	1.50 *	1.938 (0.701)	1.955 (0.746)	0.26	1.970 (0.715)	1.923 (0.752)	−0.78
Farm experience	19.179 (12.821)	18.881 (13.060)	−0.26	18.891 (12.458)	19.019 (0.658)	0.11	17.665 (12.229)	20.681 (13.719)	2.84 **
Age	43.839 (7.398)	43.887 (6.515)	0.08	43.75 (7.586)	43.931 (6.402)	0.30	43.755 (6.600)	44.022 (7.073)	0.48
Asset ownership	2.280 (0.949)	2.410 (0.976)	1.54 *	2.561 (1.077)	2.275 (0.045)	−3.43 ***	2.381 (0.923)	2.352 (1.026)	−0.35
Household size	6.005 (1.886)	5.708 (1.751)	−1.90 *	5.796 (1.919)	5.807 (1.741)	0.07	5.912 (1.770)	5.663 (1.830)	−1.68 *
Farm size	12.953 (5.676)	12.899 (6.096)	−0.10	12.786 (5.779)	12.980 (6.051)	0.37	12.820 (6.056)	13.042 (5.840)	0.45
Access to credit	1.653 (0.477)	1.548 (0.498)	−2.43 **	1.631 (0.484)	1.558 (0.497)	−1.69 *	1.585 (0.494)	1.579 (0.495)	−0.15
Extension officer	2.420 (0.997)	2.000 (1.024)	−4.73 ***	1.974 (0.974)	2.213 (0.052)	2.66 **	2.121 (1.085)	2.153 (0.965)	0.38
Household income	7.241 (0.609)	7.267 (0.527)	0.50	7.278 (0.479)	7.249 (0.588)	−0.55	7.270 (0.561)	7.243 (0.548)	−0.56
Farm income	7.263 (0.632)	7.400 (0.585)	2.53 **	7.466 (0.594)	7.308 (0.601)	−2.94 **	7.399 (0.590)	7.304 (0.615)	−1.87 *
Off-farm income	6.346 (0.769)	6.384 (0.776)	0.54	6.514 (0.321)	6.315 (0.885)	−2.69 **	6.355 (0.744)	6.392 (0.809)	0.54

Note: Standard errors are presented in parentheses; *, **, and *** represent significance level at 10, 5, and 1 per cent, respectively.

**Table 5:** Determinants of climate-smart agricultural practices.

Variables	Adoption of Irrigation		Buying of Crop Insurance		Adoption of Organic Fertilizer	
	Coeff.	Margins	Coeff.	Margins	Coeff.	Margins
Gender	0.016 (0.123)	0.219	0.137 (0.119)	0.216	0.361 (0.211)	0.291 *
Education	−0.231 (0.119)	−0.553 *	−0.188 (0.119)	−0.523 *	0.123 (0.125)	0.150
Farm experience	−0.002 (0.004)	−0.040	−0.002 (0.004)	−0.036	−0.015 (0.007)	−0.199 *
Age	−0.115 (0.042)	−0.098	−0.134 (0.071)	−0.328 *	−0.123 (0.060)	−0.303 *
Asset ownership	−0.140 (0.066)	−0.388 **	0.114 (0.059)	0.297 *	−0.049 (0.139)	−0.068
Household size	0.056 (0.032)	0.019	0.006 (0.033)	0.042	0.109 (0.051)	0.400 *
Farm size	−0.004 (0.007)	−0.064	−0.007 (0.007)	−0.061	−0.023 (0.010)	−0.131 *
Access to credit	0.230 (0.117)	0.407 *	0.253 (0.119)	0.453 *	−0.105 (0.186)	−0.098
Extension officer	0.146 (0.057)	−0.405 *	−0.097 (0.060)	−0.251	0.463 (0.176)	0.428 **
Farm membership	0.548 (0.148)	0.385 ***	0.502 (0.124)	0.774 ***	0.498 (0.243)	0.472 *
_cons	−1.525 (0.602)		−1.564 (0.672)		0.465 (0.906)	
Log likelihood	−343.749		−336.278		−142.727	
Wald chi2 (13)	33.28		50.36		30.67	
Prob > chi2	0.002		0.000		0.006	
Pseudo R2	0.048		0.069		0.106	
Observations	577		577		577	

Note: Standard errors are presented in parentheses; *, **, and *** represent significance level at 10, 5, and 1 per cent, respectively.

**Table 6:** Endogenous switch regression model estimation on adoption of irrigation system on income levels of cocoa farmers.

Variables	Adoption of Irrigation System								
	First Stage Selection Equation	Adopters	Non-Adopters	First Stage Selection Equation	Adopters	Non-Adopters	First Stage Selection Equation	Adopters	Non-Adopters
		Household Income			Farm Income			Off-Farm Income	
Gender	0.020 (0.129)	−0.001 (0.096)	0.086 0.064)	0.011 (0.119)	−0.180 (0.125)	0.014 *** (0.005)	−0.006 (0.129)	−0.043 (0.117)	−0.045 (0.085)
Education	−0.118 ** (0.045)	−0.065 (0.072)	−0.008 (0.041)	−0.080 * (0.043)	−0.054 (0.090)	0.118 * (0.058)	−0.125 ** (0.046)	0.212 * (0.088)	0.094 * (0.054)
Farm experience	0.001 (0.005)	0.002 (0.004)	−0.005 * (0.002)	−0.001 (0.005)	−0.003 0.005)	−0.001 (0.002)	−0.003 (0.005)	−0.002 (0.005)	0.006 * (0.003)
Age	−0.003 (0.009)	−0.004 (0.006)	−0.004 (0.004)	−0.005 (0.008)	0.002 (0.008)	−0.001 (0.005)	−0.006 (0.009)	−0.003 (0.008)	0.001 (0.006)
Asset ownership	−0.122 * (0.067)	−0.114 * (0.052)	0.044 (0.035)	−0.116 * (0.061)	−0.114 * (0.064)	0.135 *** (0.034)	−0.115 * 0.068)	0.023 (0.068)	0.026 (0.046)
Household size	0.045 (0.034)	0.050 * (0.026)	0.065 * (0.034)	0.063 * (0.031)	0.052 (0.033)	0.005 (0.018)	0.059 * (0.035)	0.001 (0.034)	−0.030 (0.024)
Farm size	−0.004 (0.007)	0.011 * (0.005)	0.003 (0.003)	−0.002 (0.006)	−0.012 * (0.007)	0.003 (0.003)	−0.008 (0.007)	0.017 * (0.007)	0.025 *** (0.004)
Access to credit	0.124 (0.127)	0.191 * (0.077)	0.053 (0.063)	0.137 (0.119)	0.206 * (0.085)	−0.113 * (0.066)	0.156 (0.128)	0.217 * (0.102)	0.028 (0.086)
Extension Officer	0.149 * (0.062)	−0.012 (0.050)	−0.045 * (0.048)	0.141 * (0.057)	0.104 * (0.062)	−0.029 (0.035)	0.158 * (0.063)	0.002 (0.062)	0.100 ** (0.031)
Farm Org membership	0.517 *** (0.147)			0.387 *** (0.096)			0.642 *** (0.680)		
_cons	−1.464 * (0.652)	6.810 *** (0.556)	7.343 *** (0.300)	−1.503 * (0.610)	6.321 *** (0.680)	7.395 *** (0.320)	−1.658 * (0.680)	6.147 *** (0.742)	6.599 *** (0.535)
/lns1	−0.539 *** (0.062)			−0.007 (0.092)			−0.337 *** (0.061)		
/lns2	−0.660 *** (0.044)			−0.560 *** (0.057)			−0.370 *** (0.049)		
/r1	0.134 (0.328)			1.883 *** (0.292)			−0.105 (0.414)		
/r2	−0.125 (0.362)			0.309 (0.315)			0.166 (0.401)		
LR test	Chi2 (1) = 81.82			Chi2 (1) = 52.22			Chi2 (1) = 84.70		
	Prob > chi2 = 0.000			Prob > chi2 = 0.000			Prob > chi2 = 0.000		
Observation	503			553			509		

Note: All outcome variables are in log-transformed forms. Standard errors are presented in parentheses; *, **, and *** represent significance level at 10, 5, and 1 per cent, respectively.

**Table 7:** Endogenous switch regression model estimation on adoption of crop insurance on income levels of cocoa farmers.

Variables	Buying of Crop Insurance								
	First Stage Selection Equation	Adopters	Non-Adopters	First Stage Selection Equation	Adopters	Non-Adopters	First Stage Selection Equation	Adopters	Non-Adopters
		Household Income			Farm Income			Off-Farm Income	
Gender	−0.006 (0.127)	−0.030 (0.094)	0.079 (0.062)	−0.018 (0.118)	−0.211 * (0.122)	0.022 (0.063)	−0.046 (0.128)	0.007 (0.121)	−0.102 (0.090)
Education	−0.122 ** (0.045)	−0.056 (0.054)	−0.096 * (0.058)	−0.087 * (0.042)	−0.103 (0.070)	−0.106 * (0.042)	−0.130 ** (0.046)	−0.045 (0.056)	−0.071 (0.053)
Farm experience	0.015 (0.010)	−0.005 (0.008)	0.008 * (0.005)	0.012 (0.009)	0.018 * (0.010)	−0.003 (0.005)	0.020 * (0.010)	0.158 * (0.083)	0.001 (0.007)
Age	0.001 (0.009)	−0.002 (0.006)	−0.004 (0.004)	−0.003 (0.008)	0.013 * (0.005)	−0.004 (0.005)	−0.004 (0.009)	−0.001 (0.005)	0.002 (0.006)
Asset ownership	−0.120 * (0.067)	−0.104 * (0.052)	0.039(0.035)	−0.112 * (0.061)	0.089 (0.062)	0.119 *** (0.034)	−0.102 (0.068)	−0.002 (0.006)	0.068 (0.049)
Household size	0.050 (0.033)	0.060 * (0.026)	0.008 (0.017)	0.066 * (0.031)	0.057 * (0.032)	0.009 (0.017)	0.059 * (0.034)	0.005 (0.042)	−0.025 (0.025)
Farm size	−0.008 (0.011)	−0.003 (0.009)	−0.009 * (0.005)	−0.003 (0.010)	0.011 (0.011)	−0.006 (0.005)	0.004 (0.010)	−0.001 (0.011)	0.005 (0.007)
Access to credit	0.107 (0.125)	0.084 (0.097)	0.206 * (0.085)	0.128 (0.117)	−0.064 (0.122)	−0.130 * (0.063)	0.157 (0.126)	0.261 * (0.131)	−0.117 (0.089)
Extension Officer	0.155 * (0.062)	0.103 * (0.045)	−0.060 * (0.035)	0.139 * (0.057)	0.203 ** (0.062)	−0.068 * (0.038)	0.144 * (0.063)	−0.176 * (0.091)	0.038 (0.049)
Farm Org membership	0.515 *** (0.146)			0.395 *** (0.101)			0.638 *** (0.148)		
_cons	−1.980 ** (0.662)	7.010 *** (0.609)	7.282 *** (0.301)	−1.833 ** (0.621)	6.294 *** (0.695)	7.534 *** (0.316)	−2.198 ** (0.678)	6.375*** (0.768)	7.013 *** (0.451)
/lns1	−0.532 *** (0.061)			−0.016 (0.085)			−0.290 *** (0.059)		
/lns2	−0.659 *** (0.047)			−0.569 *** (0.045)			−0.285 *** (0.040)		
/r1	0.126 (0.330)			1.847 *** (0.242)			0.049 (0.763)		
/r2	−0.169 (0.347)			0.203 (0.286)			−0.111 (0.251)		
LR test	Chi2 (1) = 84.21			Chi2 (1) = 57.98			Chi2 (1) = 86.65		
	Prob > chi2 = 0.000			Prob > chi2 = 0.000			Prob > chi2 = 0.000		
Observation	503			553			509		

Note: All outcome variables are in log-transformed forms. Standard errors are presented in parentheses; *, **, and *** represent significance level at 10, 5, and 1 per cent, respectively.

**Table 8:** Endogenous switch regression model estimation on adoption of organic fertilizer on income levels of cocoa farmers.

Variables	Adoption of Organic Fertilizer								
	First Stage Selection Equation	Adopters	Non-Adopters	First Stage Selection Equation	Adopters	Non-Adopters	First Stage Selection Equation	Adopters	Non-Adopters
		Household Income			Farm Income			Off-Farm Income	
Gender	0.096 (0.124)	0.103 (0.071)	−0.154 * (0.071)	−0.017 (0.113)	−0.147 * (0.071)	0.028 (0.103)	0.064 (0.122)	−0.113 (0.093)	0.073 (0.114)
Education	−0.013 (0.042)	0.202 * (0.085)	−0.072 (0.050)	−0.021 (0.039)	−0.024 (0.048)	0.032 (0.068)	−0.054 (0.042)	0.126 * (0.061)	0.169 * (0.073)
Farm experience	−0.012 * (0.005)	−0.002 (0.003)	0.017 *** (0.005)	−0.010 * (0.004)	0.018 * (0.010)	−0.005 (0.004)	−0.012 (0.005)	0.022 * (0.012)	−0.005 (0.004)
Age	−0.130 * (0.063)	−0.005 (0.005)	0.023 * (0.010)	0.004 (0.008)	0.013 * (0.005)	−0.005 (0.007)	−0.004 (0.009)	−0.006 (0.006)	0.001 (0.008)
Asset ownership	0.021 (0.064)	0.129 * (0.073)	−0.041 (0.041)	0.022 (0.059)	0.076 * (0.037)	0.062 (0.052)	0.051 (0.065)	0.076 (0.049)	−0.023 (0.058)
Household size	0.056 * (0.032)	0.027 (0.019)	0.017 (0.023)	0.060 * (0.030)	−0.057 * (0.032)	−0.026 (0.027)	0.062 * (0.032)	−0.033 (0.029)	−0.004 (0.030)
Farm size	−0.009 (0.006)	0.001 * (0.004)	0.011 * (0.006)	−0.008 (0.006)	0.002 (0.004)	0.004 (0.005)	0.008 (0.007)	0.018 ** (0.006)	0.026 *** (0.006)
Access to credit	−0.017 (0.122)	0.213 * (0.089)	0.135 * (0.078)	−0.011 (0.112)	−0.211 * (0.122)	0.203 ** (0.062)	0.126 * (0.071)	−0.046 (0.087)	0.204 * (0.099)
Extension Officer	0.010 (0.059)	−0.048 (0.033)	−0.211 * (0.121)	−0.176 * (0.091)	−0.051 (0.033)	−0.043 (0.052)	0.158 * (0.083)	0.229 ** (0.076)	−0.187 * (0.109)
Farm Org membership	0.237 * (0.123)			0.261 * (0.131)			0.298 ** (0.122)		
_cons	−2.345 *** (0.685)	6.943 *** (0.390)	7.370 *** (0.462)	−1.735 ** (0.654)	7.954 *** (0.384)	7.534 *** (0.316)	−1.136 *** (0.541)	6.682 *** (0.546)	7.126 *** (0.568)
/lns1	−0.599 *** (0.053)			−0.523 *** (0.085)			−0.371 *** (0.058)		
/lns2	−0.617 *** (0.056)			−0.102 (0.074)			−0.131 (0.095)		
/r1	0.157 (0.351)			0.447 (0.323)			−0.097 (0.702)		
/r2	−0.061 (0.712)			−1.631 *** (0.205)			0.968 *** (0.227)		
LR test	Chi2 (1) = 98.24			Chi2 (1) = 53.16			Chi2 (1) = 95.36		
	Prob > chi2 = 0.000			Prob > chi2 = 0.000			Prob > chi2 = 0.000		
Observation	503			553			509		

Note: All outcome variables are in log-transformed forms. Standard errors are presented in parentheses; *, **, and *** represent significance level at 10, 5, and 1 per cent, respectively.

**Table 9:** Telasso average treatment effect of climate-smart agricultural practices on farmers’ income.

**Impact of Irrigation**						
Average Treatment Effect	Household Income		Farm Income		Off-farm Income	
	Coeff	Robust Std. Err	Coeff	Robust Std. Err	Coeff	Robust Std. Err
1 vs. 0	0.086 *	0.036	0.111 *	0.060	0.017	0.072
0	6.495 ***	0.018	7.388 ***	0.031	6.369 ***	0.042
**Impact of Crop Insurance**						
Average Treatment Effect	Coeff	Robust Std. Err	Coeff	Robust Std. Err	Coeff	Robust Std. Err
1 vs. 0	0.159 **	0.050	0.142 *	0.059	0.179 **	0.052
0	6.319 ***	0.043	7.322 ***	0.471	6.329 ***	0.044
**Impact of Organic Fertilizer**						
Average Treatment Effect	Coeff	Robust Std. Err	Coeff	Robust Std. Err	Coeff	Robust Std. Err
1 vs. 0	0.011	0.050	0.084	0.052	0.017	0.064
0	7.249 ***	0.037	7.308 ***	0.278 ***	6.365 ***	0.049

Note: Standard errors are presented in parentheses; *, **, and *** represent significance level at 10, 5, and 1 per cent, respectively.

**Table 10:** Nearest neighbor matching and propensity score matching algorithm average treatment effect (robust analysis).

Household Income			Farm Income		Off-Farm Income	
Adoption of irrigation						
	Coeff	Robust Std. Err	Coeff	Robust Std. Err	Coeff	Robust Std. Err
NNM ATE 1 vs. 0	0.225 *	0.112	0.217 *	0.121	0.215 *	0.120
PSM ATE 1 vs. 0	0.188 **	0.056	0.939 ***	0.238	0.385	0.298
Crop Insurance						
NNM ATE 1 vs. 0	0.297 ***	0.082	0.263 ***	0.075	0.157 **	0.065
PSM ATE 1 vs. 0	0.313 ***	0.088	0.309 ***	0.070	0.091 *	0.037
Organic Fertilizer						
NNM ATE 1 vs. 0	0.045	0.085	0.076	0.098	0.049	0.095
PSM ATE 1 vs. 0	0.110	0.084	0.156	0.099	0.122	0.088

Note: Standard errors are presented in parentheses; *, **, and *** represent significance level at 10, 5, and 1 per cent, respectively.

## Data Availability

The datasets used and/or analyzed during the current study are available from the corresponding author on reasonable request.
